# Parasitoid Complex of Fall Armyworm, *Spodoptera frugiperda*, in Ghana and Benin

**DOI:** 10.3390/insects11020068

**Published:** 2020-01-21

**Authors:** Lakpo Koku Agboyi, Georg Goergen, Patrick Beseh, Samuel Adjei Mensah, Victor Attuquaye Clottey, Raymond Glikpo, Alan Buddie, Giovanni Cafà, Lisa Offord, Roger Day, Ivan Rwomushana, Marc Kenis

**Affiliations:** 1 CABI, P.O. Box CT 8630, Cantonments, Accra GA 0376800, Ghana; samuelmensah1a@gmail.com (S.A.M.); v.clottey@cabi.org (V.A.C.); 2 International Institute of Tropical Agriculture (IITA), 08 BP 0932 Tri Postal, Cotonou, Benin; g.goergen@cgiar.org; 3 Plant Protection and Regulatory Services Directorate (PPRSD), P.O. Box M37, Accra 00495426, Ghana; pkbeseh@gmail.com (P.B.); glikporaymond@yahoo.com (R.G.); 4 CABI, Bakeham Lane, Egham TW20 9TY, UK; a.buddie@cabi.org (A.B.); g.cafa@cabi.org (G.C.); l.offord@cabi.org (L.O.); 5 CABI, 673 Limuru Road, Muthaiga, P.O. Box 633, Nairobi 00621, Kenya; r.day@cabi.org (R.D.); i.rwomushana@cabi.org (I.R.); 6 CABI, 1 Rue des Grillons, 2800 Delémont, Switzerland; m.kenis@cabi.org

**Keywords:** biological control, *Chelonus bifoveolatus*, *Coccygidum luteum*, *Telenomus remus*, *Trichogramma*, West Africa

## Abstract

The fall armyworm, *Spodoptera frugiperda*, a moth originating from the American continent, has recently invaded most African countries, where it is seriously threatening food security as a pest of cereals. The current management methods rely heavily on the use of synthetic insecticides but there is a need for more sustainable control methods, including biological control. Surveys were conducted in two West African countries, Ghana and Benin, to determine the native parasitoid complex and assess parasitism rates of *S. frugiperda*. Samples of *S. frugiperda* eggs and larvae were collected in maize fields located in 56 and 90 localities of Ghana and Benin, respectively, from July 2018 to July 2019. Ten species were found parasitizing the pest, including two egg parasitoids, one egg–larval, five larval and two larval–pupal parasitoids. The two most abundant parasitoids in both countries were two Braconidae: the egg-larval parasitoid *Chelonus bifoveolatus* and the larval parasitoid *Coccygidum luteum.* Parasitism rates were determined in three Ghanaian regions and averages varied from 0% to 75% between sites and from 5% to 38% between regions. These data provide an important baseline for the development of various biological control options. The two egg parasitoids, *Telenomus remus* and *Trichogramma* sp. can be used in augmentative biological control and investigations should be conducted to assess how cultural practices can enhance the action of the main parasitoids, *C. luteum* and *Ch. bifoveolatus,* in the field. Understanding the parasitoid complex of *S. frugiperda* in Africa is also necessary before any development of classical biological controls involving the introduction of parasitoids from the Americas.

## 1. Introduction

The fall armyworm, *Spodoptera frugiperda* (J.E. Smith) (Lepidoptera: Noctuidae), is a polyphagous pest originating from tropical and subtropical America, where it undergoes long-distance annual migrations [1]. Known mostly as a major pest of maize and other cereals [2], it has recently invaded most maize agroecosystems in Africa and it is now rapidly spreading in Asia [3,4,5]. In West Africa, *S. frugiperda* was first reported in Benin, Nigeria and Togo in 2016 [5,6] and one year later in Ghana [7]. The species does not diapause, and the favourable climatic conditions in most African countries allow the pest to complete several generations per year, wherever host plants are available, including off-season and irrigated crops [8]. Left unmanaged and in the absence of natural antagonists, *S. frugiperda* has been reported to cause significant yield losses in maize and other crops in Africa [9,10].

In Africa, the immediate response for the management of this pest has focused primarily on synthetic insecticides, many of which are, as yet, unregistered for use on this new threat [4,11]. Although there is a deliberate effort in some countries to develop and promote integrated approaches for the control of *S. frugiperda*, many farmers still rely on chemical insecticides [4,12,13]. This practice is not only costly to the farmer and economically unsustainable for resource-poor farmers, but it poses a risk to human health, can cause environmental pollution, and can favour the development of insecticide resistance, as found in the Americas [14,15]. The frequent and improper use of pesticides could also disrupt the effectiveness of other integrated pest management measures targeted at other pests, such as stemborers, in maize cropping systems. The sustainable management of this invasive pest, therefore, requires the development and dissemination of ecologically friendly crop protection methods. Biopesticides could be an option, but a recent analysis of the availability of biopesticides in Africa revealed that in many countries few active ingredients are registered and most of them are not yet registered against *S. frugiperda* [16]. Methods such as the use of botanicals have recently been suggested as another suitable option [10,16,17], but their wider use is constrained by availability and cost. Therefore, biological control approaches that exploit the use of parasitoids and predators become viable alternatives for the management of this pest, particularly among resource-constrained smallholder farmers.

Parasitoids and predators can be used against *S. frugiperda* through the augmentation or conservation of natural enemies that already occur in the target environment. However, there is presently limited available information on the natural enemies of *S. frugiperda* in Africa. Some authors studied the parasitoid complex of the pest in Ethiopia, Kenya, Tanzania [18,19] and Senegal [20]. In addition, the platygasterid egg parasitoid *Telenomus remus* Dixon was recorded in Côte d’Ivoire, Benin and Niger [21] and the biology of the braconid larval parasitoid (*Coccygidium luteum* Brullé) has recently been studied in Ghana [22]. There are many known natural enemies of *S. frugiperda* from North, Central and South America that play an important role in the natural suppression of this pest in its area of origin (e.g., [23,24,25,26,27,28]) and could potentially be considered for introduction into Africa. Parasitoids are probably better candidates for introduction since at least some of them may be rather specific, whereas all predators attacking *S. frugiperda* are known to be polyphagous [8]. However, the importation of American parasitoids into Africa also requires preliminary studies of the parasitoid complex of the moth in the invaded area, to assess potential gaps in the complex that could be filled with exotic species, as well as to assess possible competitive interaction between exotic and native parasitoids [29].

The present study was undertaken to provide an inventory of the parasitoid complex of *S. frugiperda* in two West African countries, Ghana and Benin, in order to evaluate their potential use as biological control agents.

## 2. Materials and Methods

### 2.1. Surveys in Ghana

In Ghana, samples were collected in 56 localities from nine regions, from the north to the south of the country. Supplementary Table S1 provides details of the collection sites with a description of the ecological zone. The collections were conducted during the maize growing seasons from July to November 2018, in maize fields that had not been previously treated with insecticides. The samples collected from the Eastern, Volta and Central regions were used to provide quantitative data on parasitism rates, whereas the samples from the other six regions were used to provide qualitative information on the presence of a given parasitoid on *S. frugiperda* in a specific region.

Different larval stages of *S. frugiperda* and egg masses were collected randomly and carried to the biological control laboratory of the Plant Protection and Regulatory Services Directorate of Ghana in Accra. The egg masses were collected with a piece of maize leaf on which they were found and placed in plastic vials with humid paper. Some larval samples were brought in bulk but, to avoid cannibalism, in the collections used for quantitative analyses, each larva was kept separately in an aerated plastic disposable cup (80 mL) containing a piece of tissue paper. Larvae and eggs were kept in the laboratory at an ambient temperature of 26–30 °C, 70%–90% relative humidity. Leaves collected from 3–4-week old untreated maize plants grown in a greenhouse were used to feed the larvae individually in their cups. The maize leaves were changed every two days until the emergence of a parasitoid adult or a *S. frugiperda* moth. Egg masses were kept individually until hatching or parasitoid emergence, in aerated plastic cups (650 mL) also containing a piece of dry tissue paper and untreated maize leaves. The data on the development of *S. frugiperda* and the emergence of parasitoids were collected every two days. All parasitoids species that emerged from the samples were conserved in 99% alcohol for morphological and molecular identifications.

In addition, a field collection of *S. frugiperda* egg masses was carried out on 30 July 2019 at three farms in Somanya, Eastern Province, where eggs were particularly abundant. The egg masses were collected and processed as described above. 

### 2.2. Surveys in Benin

Rainfall in Benin follows a unimodal pattern in the northern part of the country (from June to September) and a bimodal pattern in the southern regions (from March to July and September to November). Thus, to meet the maize growing season across the whole country, it was decided to organize two separate field surveys. A first survey was conducted from end of July to the beginning of August 2018 for northern Benin and a second in mid-October 2018 for the southern regions stretching from Bohicon in the centre to Cotonou at the littoral. During each of the surveys, stops were made at 20 km intervals following a North-South transect to scout for egg masses and immatures of *S. frugiperda* using the standard procedure laid out in [8]. In total, 90 stops were made at different locations, enabling visits to 102 maize fields (Supplementary Table S1). Samples of detected egg masses and immatures were brought back to the IITA station-Benin for laboratory rearing according to the method described above.

In addition to these surveys, the egg parasitism of *S. frugiperda* was determined by collecting and rearing egg masses that were sampled every two days during the entire month of June 2019 on young maize plants in fields that had been prepared at the IITA station at Calavi. The collected egg batches were held in 8 mL plastic vials sealed with cotton wool and maintained at an ambient temperature for insect emergence. The vials were regularly monitored for 15 days. Representative specimens were used for barcoding and voucher samples of adult insects from all recovered species were deposited in the reference collection of the Biodiversity Centre at the IITA station, Benin.

### 2.3. Morphological and Molecular Identification

All parasitoids obtained during these studies were examined and morphologically identified using various identification keys and taxonomic descriptions (e.g., [30,31,32,33]) by two authors (GG and MK) and collections at CABI and IITA-Benin for specimen comparisons. Some representative specimens were brought to the Natural History Museum London for comparison with the collection holdings and were identified with the assistance of group specialists. In addition, samples of each species were subjected to molecular analyses using the mtDNA barcode gene in order to compare them with existing barcode datasets. To obtain barcodes (around 600 bp of the mitochondrial gene (mtDNA) cytochrome c oxidase subunit 1 (COI)) from the samples, we followed the protocols described in [21]. The sequences obtained in the present study were compared with authenticated sequences available from the Barcoding of Life Data system (BOLD; http://www.boldsystems.org/) [34] and additional sequences from the GenBank^®^ data base (http://www.ncbi.nlm.nih.gov/genbank/) [35].

### 2.4. Relative Abundance and Parasitism Rates

The relative abundance of larval parasitoids and larval parasitism rates were assessed at sites in the Eastern, Volta and Central regions in Ghana, where *S. frugiperda* larvae were sampled regularly (sites indicated by * in Supplementary Table S1). For the other collections, mortality by cannibalism and other intrinsic and extrinsic causes during transfer to the laboratory was too high to provide reliable data on parasitism.

The relative abundance of a parasitoid species (RA) was determined by calculating the number of individuals of each parasitoid species (*N_i_*) in the total number of parasitoids obtained from the sample collected (*N_t_*) and expressing this value as a percentage.

The parasitism rate (PR) was calculated as the number of a parasitoid species (or all parasitoids species for the case of total parasitism) divided by the total number of parasitoids and hosts that reached at least the pupal stage from the sample collected, expressed as a percentage. The hosts that died at the larval stage were not included in the calculation, since it could not be determined whether they were parasitized or not. No gregarious parasitoids were observed in the samples.

Parasitism rates were also estimated from samples of egg masses collected in July 2019 in Somanya in Ghana and in June 2019 at the IITA station in Calavi. The percentage of egg masses providing egg parasitoids was calculated and, in Benin, the percentage of parasitized eggs per egg mass was measured.

## 3. Results

### 3.1. Parasitoid Complex of S. frugiperda in Ghana and Benin

Ten parasitoid species were found in the two countries—eight in Ghana and nine in Benin—from which DNA barcode sequences were obtained (Table 1). 

They included two egg parasitoid species, one egg-larval parasitoid species, five larval parasitoid species, and two larva-pupal parasitoid species. *Chelonus bifoveolatus* Szépligeti and *C. luteum* were the most abundant and frequent species encountered, being present in nine and seven regions, respectively, out of the nine surveyed Ghanaian regions, and eight departments of the 10 visited departments in Benin, respectively (Table 2 and Table 3). They were collected in 23 and 16 localities, respectively, from a total of 56 localities visited in Ghana. The remaining larval parasitoid species were less frequently collected. The egg parasitoid *T. remus* was found only at three and 24 locations in Ghana and Benin, respectively. However, in Ghana, *S. frugiperda* egg masses were collected only in a part of the investigated localities and were specifically searched only at one locality (Somanya, Eastern Region).

In Ghana, in the East and Ashanti regions, the number of parasitoid species recorded was seven and five, respectively. In the Volta and Brong Ahafo regions, four parasitoid species were collected. In the five other regions, the number of parasitoid species collected ranged between one and three, with only one species being found in the Upper West region (Table 2).

### 3.2. Relative Abundance of S. frugiperda Parasitoids and Parasitism Rates

Quantitative data on *S. frugiperda* larval parasitoids assessed in selected localities of the Eastern, Volta and Central regions of Ghana are presented in Table 4 and Table 5. *Coccygidium luteum* and *Ch. bifoveolatus* were by far the most abundant parasitoid species, with a relative abundance estimated at 49% and 48% in the Eastern Region and 44% and 41% in the Volta Region, respectively (Table 4). In contrast, in the Central region, *Ch. bifoveolatus* was not present in the quantitative samples and *C. luteum* was the most abundant (69%), followed by *Charops* sp. (31%). All other larval or larval–pupal parasitoids were rare (RA < 1%) or absent in the three regions.

The highest total larval parasitism rate was observed in the Eastern region (38.8%), followed by the Volta region (10.7%) and the Central region (5.1%) (Table 4). At the species level, *C. luteum* and *Ch. bifoveolatus* caused an average of 19.3% and 18.9% parasitism, respectively, in the Eastern region. Parasitism was much lower in the Volta and Central regions (Table 4). With respect to the specific locations, the parasitism rate reached 75.0% in Somanya (Eastern region) in August 2018, with *C. luteum* and *Ch. bifoveolatus* being the main parasitoids (Table 5). Overall, the larval parasitism rates decreased from August to November 2018.

In Ghana, 174 egg masses were recovered from farm surveys carried out at three sites in the Eastern Region in July 2019, and 45 of them (25.9%) were parasitized by *T. remus*. In Benin, a total of 145 egg masses were recovered in maize fields following 33 days of sampling. Egg parasitism was observed in 14.5% of the reared egg batches, from which emerged an average of 42.1 parasitoids—i.e., a parasitism rate of 41.9% within attacked egg masses. Except for two occasions, where *Trichogramma* sp. and *T. remus* hatched together from the same egg mass, field parasitism in Benin led solely to the emergence of *T. remus*.

## 4. Discussion

### 4.1. Parasitoid Complex and Parasitism of S. frugiperda in Ghana and Benin

Ten parasitoid species attacking the eggs and larvae of *S. frugiperda* were found in Ghana and Benin. In similar surveys carried out in Ethiopia, Kenya and Tanzania, seven parasitoid species were collected [18,19]. The two main parasitoids of *S. frugiperda* in Ghana and Benin are two braconids, the larval parasitoid *C. luteum* and the egg–larval parasitoid *Ch. bifoveolatus*. Based on photos provided in [20], these were likely also the two larval parasitoids recovered from *S. frugiperda* in Senegal, even though the authors identified them as *Campoletis* sp. and *Chelonus* sp., respectively. *Coccygidium luteum* was also found to attack fall armyworms in Ethiopia, Kenya and Tanzania [18,19]. In contrast, two different *Chelonus* species were found in West and East Africa. *Chelonus curvimaculatus* Cameron was obtained in Kenya [18,19] whereas *Ch. bifoveolatus*, a comparatively larger species, is prevalent in Ghana and Benin. Moreover, numerous previous records of this parasitoid are reported from Burkina Faso, Cameroon, Chad, DR Congo, Madagascar, Nigeria, Sudan, Togo and Tanzania from *Spodoptera* spp. [30,37,38]. In Benin, *Ch. bifoveolatus* has occasionally been recovered from caterpillars of *Spodoptera exigua* (Hübner) feeding on onions in peri-urban vegetable gardens along the coast [39]. Interestingly, the barcode data indicate a > 99% congruence with the *Chelonus* species from Zimbabwe and Kenya but also from South Asia and Polynesia. The microgasterid *Cotesia icipe* was recorded in very low numbers in Ghana and Benin. By contrast, in Ethiopia, Kenya and Tanzania, it has become the most abundant larval parasitoid of *S. frugiperda* [18,19] following its recent description from specimens obtained from *S. exigua* and *Spodoptera littoralis* (Boisduval) [33]. Since these two latter moth species are widely spread throughout sub-Saharan Africa (SSA), the reason for the sporadic occurrence of *C. icipe* on *S. frugiperda* in West Africa calls for further research.

The species of *Charops* reared from *S. frugiperda* in West Africa [this study] and East Africa [18,19] is possibly the same. However, the taxon cited in [18,19], *Charops ater* Szépligeti, is confusing [40]. Comparisons with the descriptions and type specimens of *C. ater* and other available African *Charops* species suggest that the species obtained from *S. frugiperda* in this study is likely undescribed. Until it is formally described, the taxon can be characterized by its barcode (Genbank accession numbers: MN900729; MN900742).

The tachinid *D. quadrizonula* collected in low numbers during our surveys in Ghana and Benin is closely related to the species *Palexorista zonata* (Curran) (= *Drino imberbis* [41]) found on *S. frugiperda* in Ethiopia and Kenya by [18,19], because *Palexorista* is a subgenus of *Drino,* according to the most recent classification of the Tachinidae of the Afrotropical region [42]. *Drino quadrizonula* is a widespread species in sub-Saharan Africa, and is known to parasitize a variety of moth larval hosts belonging to several families, though most records originate from Noctuidae, including several other *Spodoptera* spp. [43].

Three other native parasitoid species have adopted fall armyworm larvae as a new host and are here reported for the first time for Africa. Among these, four specimens of *Pristomerus pallidus* were collected in coastal Benin. Our molecular analyses showed that it is the same species as the one attacking the millet head miner, *Heliocheilus albipunctella* De Joannis, in Senegal [36]. Two larval–pupal parasitoids, *Metopius discolor* and *Meteoridea* cf. *testacea*, were reared in very low numbers. *Metopius discolor* is widespread in tropical Africa and commonly recorded from *Spodoptera exempta* (Walker), the African armyworm, which also attacks cereals [30,32]. *Meteoridea* is a rare genus, with only two known African species. The identification of the *Meteoridea* species is tentative and needs confirmation. The literature records indicate that a species of *Meteoridea* has already been reared from *S. exempta* in Tanzania [44] but the genus appears to be polyphagous as it can also develop in the pupae of the crambid *Haritalodes derogata* (Fabricius) in Africa [45].

Finally, two parasitoids were collected from eggs of *S. frugiperda* during our surveys. *Telenomus remus* is common and was recently found in other African countries [19,21]. Only three egg masses were found attacked by *Trichogramma* sp. in Benin and the specimens could not be identified to the species level. *Trichogramma chelonis* Ishii was recently reared from *S. frugiperda* in Kenya [19]; however, the barcode of the Benin specimens did not correspond to *T. chelonis* but to a *Trichogramma* sp. collected in Mali from egg batches of the noctuid moth *Helicoverpa armigera* (Barcode Index Number Registry For BOLD: ADS7997).

The field parasitism rates were very variable but sometimes surprisingly high for a newly invasive species. Larval parasitism of up to 75% was observed at one site. However, many collections did not result in any parasitoids. The new associations of native parasitoids with *S. frugiperda* might be attributable to the occurrence of several other *Spodoptera* species in West Africa, such as *S. exempta*, *S. exigua* and *S. littoralis*. Interestingly, there is only very little overlap between the parasitoid guild of *S. frugiperda* and the one associated with cereal stem borers such as *Eldana saccharina* Walker, *Sesamia* spp., *Busseola fusca* (Fuller), and *Chilo aleniellus* (Strand) [46,47,48,49,50,51].

In Ghana, larval parasitism was higher, and the parasitoid complex was richer, in the south than in the north. For example, *Charops* sp. was common in the south but not collected in the north. This could be due to ecological factors such as vegetation and rain patterns. In the south, the long periods of rains and higher plant and insect richness may offer better conditions for parasitoid diversity and proliferation.

Egg parasitism by *T. remus* was low, with 25.9% of the egg masses parasitized at one location in Ghana and 14.5% at another location in Benin. In Kenya and Tanzania, authors [19] mention egg parasitism rates by *T. remus* above 50%. However, it must be noted that eggs parasitized by *T. remus* remain at least four times longer in the field than unparasitized eggs because the duration of the egg stage is only two to three days during the warm summer months [16] whereas the development of *T. remus* at 25 °C lasts 12 to 13 days [52]. Furthermore, parasitized eggs are dark, and thus more visible than unparasitized ones. Therefore, casual collections such as those carried out in Ghana (this study), Kenya and Tanzania [19] probably overestimate egg parasitism rates. In contrast, regular examinations of the same plants such as those conducted in Benin are more likely to provide more accurate parasitism rates.

### 4.2. Prospects for Biological Control

The presence of several parasitoids that can reach moderate or high parasitism rates under some conditions provides important information for the development of biological controls. Some parasitoids could be used for augmentative releases. The prospects for *T. remus*, which is already used in Latin America against *S. frugiperda* [53,54,55], have been elaborated in detail by [21]. This Asian species, deliberately introduced into the Americas, has been recently found in several African countries. Moreover, the finding of a *Trichogramma* sp. on *S. frugiperda* in Africa adds to the arsenal of natural enemies that could be exploited for control of this pest. *Trichogramma* spp. are commonly used in cereal crops for the augmentative biological control of *S. frugiperda* in the Americas [56,57]. Larval parasitoids are, in general, more difficult to use in open-field augmentative biological control than egg parasitoids because of the difficulty to produce them in high quantity for mass releases. However, there are exceptions, such as *Habrobracon hebetor* Say, which is used against *H. albipunctella* in millet fields in the Sahel region [58]. Some authors [22] discussed the potential of using *C. luteum* in augmentative biological control. The challenge of using a larval parasitoid of *S. frugiperda* in augmentative biological control will be to find alternate rearing hosts for mass production, since *S. frugiperda* is not suitable for the mass production of larval parasitoids due to the cannibalistic behaviour of the larvae [16].

The fact that parasitoids such as *Ch. bifoveolatus, C. luteum* and *T. remus* can reach high rates of parasitism opens possibilities for conservation biological control. Cultural practices are known to enhance the parasitism of crop pests and various agroecological options may be used against *S. frugiperda*, such as replacing synthetic insecticides by botanicals or biopesticides, considering intercropping and other habitat management methods, or planting field margins [59]. However, the effect of these practices on parasitism of *S. frugiperda* remains to be studied.

Finally, the information on native parasitoids in West Africa gathered during this study may also help in selecting parasitoids for introduction from the Americas. For example, the abundance and high frequency of *Ch. bifoveolatus* in West Africa suggests that the introduction of *Chelonus insularis* Cresson, probably the most widespread and frequently cited parasitoid of *S. frugiperda* in its native range [25], may not be required, since it would directly compete with the local *Ch. bifoveolatus.* Their respective performance on *S. frugiperda* should be compared to assess whether *C. insularis* would show advantages compared to *C. bivoveolatus.* In contrast, the low population and incidence of *C. icipe* in the region and the absence of other microgasterine parasitoids may advocate for the introduction of *Cotesia marginiventris* (Cresson) one of the most important larval parasitoid of *S. frugiperda* on the American continent, known for a relatively good tolerance to pesticides in maize fields in the USA [28]. However, this parasitoid is recorded as being polyphagous [36] and potential non-target effects would need to be properly assessed before introduction into Africa. Parasitoids such as *Eiphosoma* spp. or *Aleiodes* spp., which are among the most specific parasitoids of *S. frugiperda* in the Americas [25,60] do not yet have congeneric species attacking the pest in Africa and may thus be considered better options for introduction in Africa.

## Figures and Tables

**Table 1:** Parasitoid species emerged from *S. frugiperda* eggs and larvae collected in maize farms in Ghana (GH) and Benin (BE).

Order, Family and Species	Country	Host Stage Attacked/Killed	Barcoding Results: GenBank Accession Number; Closest Species or Genus
Hymenoptera: Platygastridae			
*Telenomus remus* Dixon	GH, BE	Egg	MN900731, MN900732; 100% similar to *T. remus* in [21]
Hymenoptera: Trichogrammatidae			
*Trichogramma* sp.	BE	Egg	MN900733; 98.9% similar to a *Trichogramma* sp. on *Helicoverpa armigera* (Hübner) in Mali (unpublished data)
Hymenoptera: Braconidae			
*Chelonus bifoveolatus* Szépligeti	GH, BE	Egg–larval	MN900730, MN900734, MN900743, MN900744; > 99% similar to *Chelonus* sp. from Africa, Asia and Polynesia
*Coccygidium luteum* (Brullé)	GH, BE	Larval	MN900728, MN900739, MN900741; 99% similar to *Coccygidium* sp from Bangladesh
*Cotesia icipe* Fernandez-Triana and Fiaboe	GH, BE	Larval	MN900735; 100% similar to type specimen [33]
*Meteoridea* cf. *testacea* (Granger)	GH, BE	Larval–pupal	MN900738; 89.7% similar to *Meteoridea* sp. from Papua New Guinea
Hymenoptera: Ichneumonidae			
*Charops* sp.	GH, BE	Larval	MN900729; MN900742; 89.6% similar to *Charops cantator*
*Metopius discolor* Tosquinet	GH	Larval–pupal	MN900737, MN900740; 96.3% similar to *Metopius* sp. from South Africa
*Pristomerus pallidus* (Kriechbaumer)	BE	Larval	MN900727; 99.5% similar to *Pristomerus pallidus* from Senegal [36]
Dipt: Tachinidae			
*Drino quadrizonula* (Thomson)	GH, BE	Larval	MN907776; 99.8% similar to *Drino* sp. from Kenya

**Table 2:** Parasitoids of *Spodoptera frugiperda* in different localities of Ghana in 2018.

Region	Locality		Natural Enemies of *S. frugiperda*						
		Egg Parasitoid	Egg-Larval Parasitoid	Larval Parasitoid				Larval-Pupal Parasitoid	
		*Tel.*	*Chel.*	*Coc.*	*Cot.*	*Cha.*	*Dri.*	*Metop.*	*Meteo.*
GH-Eastern	Somanya *	+	+	+	+	+			
	Okwenya *		+					+	
	Kpong	+							
	Apese *			+					
	Apewu			+					
	Adawso		+				+		
GH-Volta	Togome *		+	+					
	Anyirawase *		+	+		+			
	Agbokope *		+	+					
	Tsito *					+			
	Dabala *			+		+			
	Matse		+		+				
	Adaklu		+		+				
GH-Central Accra	Jukwa *					+			
	Cape Coast campus *			+		+			
	Assin-Endwa					+			
	Yamoransa			+					
	Ekumfi-Edukuma		+	+		+			
GH-Greater-Accra	Adenta	+	+	+					+
GH-Ashanti	Akyeremade			+					
	Adidwan			+			+		
	Ejura farms			+	+	+	+	+	
	Breku			+					
GH-Brong Ahafo Region	Madina (Busunya road)		+						
	Dobidi Nkwanta		+	+		+	+	-	-
	Prang			+					
	Dawadawa			+					
GH-Northern	Wasipe			+					
	Sanyeri		+	+					
	Kukobila		+						
	Zangbalum		+						
	Benyunkwa			+					
GH-Upper West	Sakalu			+					
GH-Upper East	Wiaga		+	+			+		

*Tel.*: *Telenomus remus*; *Che.*: *Chelonus bifoveolatus*; *Coc.*: *Coccygidium luteum*; *Cot.*: *Cotesia icipe*; *Cha.*: *Charops* sp.; *Dri.*: *Drino quadrizonula*; *Metop.*: *Metopius discolour*; *Meteo.*: *Meteoridea* cf. *testacea*; + Parasitoid species present in the locality; only localities where parasitoids were found are listed in this table; * Sites where collections were regular and conducted properly to calculate parasitism rates.

**Table 3:** Parasitoids of *Spodoptera frugiperda* in different localities of Benin in 2018.

Region	Locality	Natural Enemies of *S. frugiperda*							
		Egg Parasitoid	Egg-Larval Parasitoid	Larval Parasitoid				Larval-Pupal Parasitoid	
		*Tel.*	*Chel.*	*Coc.*	*Cot.*	*Cha.*	*Dri.*	*Metop.*	*Meteo.*
Atacora	Natitingou range			+					
	Toukountouna			+					
Atlantique	Attogon	+							
	Calavi	+		+	+	+	+		
	Cococodji	+	+						
	Massi	+							
	Ouidah		+	+					
	Sékou		+			+			
	Sérouhé	+		+		+			
	Womey	+							+
Borgou	Bouyerou	+		+					
	Guessou		+						
	Ina			+					
	Parakou town		+			+			
	Parakou range	+							
Collines	Bante		+	+					
	Dassa					+			
	Gobe	+							
	Savalou		+						
Couffo	Aplahoué	+							
	Hagoumey	+	+						
Donga	Aoro		+	+					
	Basilla	+							
	Prékété	+		+					
Mono	Athiémé	+							
	Gandjazoumé	+							
	Grand Popo	+	+	+					+
	Hilacondji	+							
	Sègbo	+							
Ouémé	Azové	+				+			
	Azaourissè	+	+	+					
	Sémé-Kpodji	+	+						
Zou	Bohicon	+	+	+		+	+		
	Kpédékpo	+							
	Passagon		+	+			+		
	Setto	+							

*Tel.*: *Telenomus remus*; *Che.*: *Chelonus bifoveolatus*; *Coc.*: *Coccygidium luteum*; *Cot.*: *Cotesia icipe*; *Cha.*: *Charops* sp.; *Dri.*: *Drino quadrizonula*; *Metop.*: *Metopius discolour*; *Meteo.*: *Meteoridea* cf. *testacea*; + Parasitoid species present in the locality; only localities where parasitoids were found are listed in this table.

**Table 4:** Abundance and parasitism rates by egg–larval and larval parasitoids of *Spodoptera frugiperda* in the Eastern, Volta and Central regions of Ghana. Mortality rates of the collected *S. frugiperda* larvae that died but produced no parasitoids are 9.3%, 7.0% and 1.6% in the Eastern, Volta and Central regions, respectively.

Parasitoid Species	Ghana Regions								
	Eastern Region (*N* = 450)			Volta Region (*N* = 890)			Central Region (*N* = 255)		
	No. of Individuals	Relative Abundance (%)	Parasitism Rate (%)	No. of Individuals	Relative Abundance (%)	Parasitism Rate (%)	No. of Individuals	Relative Abundance (%)	Parasitism Rate (%)
*Chelonus bifoveolatus*	85	48	18.9	41	43	4.6	-		
*Cotesia icipe*	2	1	0.4	-			-		
*Charops* sp.	1	1	0.2	10	11	1.1	4	31	1.6
*Coccygidium luteum*	87	49	19.3	44	46	4.9	9	69	3.5
Total	175		38.8	95		10.7	13		5.1

*N*: Total Number of *S. frugiperda* larvae collected in maize farms in the region; - Absence of data due to absence of parasitoid species in samples used.

**Table 5:** Percentage of parasitism by egg–larval and larval parasitoids of *Spodoptera frugiperda* in selected localities in Ghana from August to November 2018. The mortality rate of the collected *S. frugiperda* larvae that died without producing a parasitoid ranged between 2.3% and 17.6%, 5.3% and 13.6% and 0% and 9.3% in August, October and November 2018, respectively.

Region	Locality	August 2018							October 2018							November 2018						
		*N* *	Parasitism Rate (%)						*N* *	Parasitism Rate (%)						*N* *	Parasitism Rate (%)					
			*Che.*	*Coc.*	*Cot.*	*Cha.*	Dead Parasitoid Larvae or Pupae	Total		*Che.*	*Coc.*	*Cot.*	*Cha.*	Dead Parasitoid Larvae or Pupae	Total		*Che.*	*Coc.*	*Cot.*	*Cha.*	Dead Parasitoid Larvae or Pupae	Total
Eastern	Somanya	160	35.6	28.1	1.3	0.6	9.4	75	74	14.9	29.7	0	0	5.4	50	147	2	6.1	0	0	4.1	12.2
	Okwenya	69	24.6	0	0	0	1.4	26	-	-	-	-	-	-	-	-	-	-	-	-	-	-
Greater-Accra	Adenta	112	7.1	0.9	0	0	6.3	14.3	-	-	-	-	-	-	-	-	-	-	-	-	-	-
Volta	Togome	34	14.7	2.9	0	0	8.8	26.5	49	0	4.1	0	0	0	4.1	102	2	7.8	0	0	2	11,8
	Anyirawase	63	12.7	20.6	0	11.1	1.6	47	-	-	-	-	-	-	-	-	-	-	-	-	-	-
	Agbokope	20	5	0	0	0	5	10	-	-	-	-	-	-	-	39	2.6	5.1	0	0	2.6	10.3
	Tsito	-	-	-	-	-	-	-	-	-	-	-	-	-	-	122	0	0	0	0.8	0	0.8
	Mafi Kpedzeglo	-	-	-	-	-	-	-	126	0	0.8	0	0	0	0.8	43	0	0	0	0	2.3	2.3
	Dabala	-	-	-	-	-	-	-	-	-	-	-	-	-	-	153	0	3.9	0	0.7	0.7	5.3
	Dzodze	-	-	-	-	-	-	-	43	32.6	0	0	0	14	46.6	-	-	-	-	-	-	-
	Asikuma	-	-	-	-	-	-	-	39	0	0	0	2.6	0	2.6	-	-	-	-	-	-	-
	Apese	-	-	-	-	-	-	-	57	0	19.3	0	0	1.8	21.1	-	-	-	-	-	-	-
Central	Jukwa	169	0	0	0	0	0	0	-	-	-	-	-	-	-	48	0	0	0	2	0	2
	Cape Coast	-	-	-	-	-	-	-	-	-	-	-	-	-	-	38	0	23.7	0	7.9	2.6	34.2

* Number of *S. frugiperda* larvae collected; *Che.*: *Chelonus bifoveolatus*; *Coc.*: *Coccygidium luteum*; *Cot.*: *Cotesia icipe*; *Cha.*: *Charops sp.*

## Supplementary Materials

The following are available online at https://www.mdpi.com/2075-4450/11/2/68/s1, Table S1: Description of the study sites in Ghana (GH) and Benin (BE).
