# Supplementary material for: Parasitoid Complex of Fall Armyworm, Spodoptera frugiperda, in Ghana and Benin
Source: Insects. 2020 Jan 21;11(2):68. doi: 10.3390/insects11020068 (PMC7073983; doi:10.3390/insects11020068)
Supplement: Supplementary file 1 [file insects-11-00068-s001.pdf]

Table S1. Description of the study sites in Ghana (GH) and Benin (BE).

Region	Locality	GPS coordinates	Ecological zone
GH-<br>Eastern Region	Somanya*	N6.062250° W0.023580°	Forest savanna transition (with mango orchards)
	Okwenya*	N6.098640° E0.027580°	
	Kpong	N6.111026° E0.049479°	
	Apese*	N5.951230° W0.014550°	
	Apewu	N5.980738° W0.231675°	
	Adawso	N6.710465° W0.523119°	
GH-<br>Volta Region	Togome*	N6.130930° E0.131060°	Semi-deciduous rainforest, mountainous area in the Northern parts of the Region
	Anyirawase*	N6.563890° E0.299120°	
	Agbokope*	N5.994710° E0.520030°	
	Tsito*	N6.510030° E0.252330°	
	Mafi Kpedzeglo*	N6.225510° E0.554310°	
	Dabala*	N6.019400° E0.659380°	
	Dzodze*	N5.948150° E0.524360°	
	Asikuma*	N6.208640° E0.106850°	
	Matse	N6.679556° E0.480250°	
	Adaklu	N6.555722° E0.489722°	
GH-<br>Central Region	Jukwa*	N5.318970° W1.375970°	Rain forest (with citrus, coconut and palm tree orchards)
	Cape Coast campus*	N5.119861° W1.289778°	
	Assin-Endwa	N5.808731° W1.297573°	
	Wurakese	N5.697360° W1.225530°	
	Yamoransa	N5.163757° W1.192219°	
	Abandze-Saltpond	N5.220110° W1.055174°	
	Ekumfi -Edukuma	N5.274603° W0.973437°	
GH-<br>Greater-Accra	Adenta	N5.689290° W0.160890°	Coastal savanna (shrubs and grassland)
GH-<br>Ashanti Region	Jamasi	N6.982061° W1.458407°	Semi-deciduous rainforest
	Akyeremade	N1.106423° W1.396105°	
	Bunuso	N7.201766° W1383281°	
	Adidwan	N7.265160° W1.379072°	
	Ejura farms	N7.386813° W1.379072°	
	Kobreso	N7.283068° W1.843468°	
	Breku	N6.602025° W0.953439°	
	Juaso	N6.604515° W1.141721°	
	Ohene Nkwanta	N6.614303° W1.262194°	
	Ejisu	N6.710787° W1.463960°	
	Ansa-Adansi	N6.096464° W1.432862°	

Table S1 (Continued)

Region	Locality	GPS coordinates	Ecological zone
GH-Brong Ahafo Region	Madina (Busunya road)	N7.645701° W1.685599°	Forest savanna transition
	Dobidi Nkwanta	N7.616618° W1.105983°	
	Praprabon	N7.834967° W0.960034°	
	Prang	N8.012416° W0.879969°	
	Dawadawa	N8.390090° W1.567950°	
GH-Northern Region	Wasipe	N8.540050° W2.204950°	Guinea savanna
	Sanyeri	N9.422040° W2.532690°	
	Kukobila	N10.113850° W0.819850°	
	Zangbalum	N9.564000° W0.976500°	
	Benyunkwa	N8.603168° W2.247666°	
GH-Upper West Region	Sakalu	N10.712858° W2.033249°	Guinea savanna
	Sankana	N10.198052° W2.587566°	
	Goli	N10.296390° W2.631374°	
	Fian	N10.384815° W2.469045°	
	Mwankuri	N10.523094° W2.506536°	
	Degya	N10.702305° W2.373259°	
	Affisi	N10.784712° W2.253585°	
	Tumu	N10.847220° W1.995391°	
GH-Upper East Region	Wiaga	N10.678139° W1.272472°	Guinea savanna
	Fumbisi	N10.433778° W1.323944°	
	Manga	N11.018472° W0.256528°	
BE-Alibori	Alfa Kouara	N11.476395° E3.081029°	Soudan savanna
	Angaradebou	N11.308378° E3.035882°	
	Bangoun	N11.624522° E3.172737°	
	Gounarou	N10.886273° E2.850106°	
	Guene	N11.743185° E3.272433°	
	Kandi	N11.173874° E2.953005°	
	Kassakou	N11.088695° E2.905833°	
	Malanville	N11.860233° E3.395781°	
	Sori	N10.686282° E2.773640°	
BE-Atacora	Dikokoré	N10.456029° E1.368444°	Soudan savanna
	Kounounko	N10.001623° E1.511536°	
	Natitingou range	N10.262371° E1.380778°	
	Natitingou town	N10.145228° E1.425260°	
	Perma	N10.110102° E1.443906°	
	Tanguieta	N10.645985° E1.257466°	
	Tanguieta range	N10.615575° E1.264815°	
	Toukountouna	N10.527919° E1.354134°	

Table S1 (Continued)

Region	Locality	GPS coordinates	Ecological zone
BE-Atlantique	Segboroué	N6.391651° E1.992318°	Guinea savanna
	Akassato	N6.492974° E2.361254°	
	Attogon	N6.715041° E2.158120°	
	Calavi*	N6.437500° E2.328330°	
	Coccharacteristicsodji	N6.388076° E2.263395°	
	Massi	N6.960470° E2.250880°	
	Ouidah	N6.370634° E2.071908°	
	Sékou	N6.611544° E2.262580°	
	Sérouhé	N6.867671° E2.240357°	
	Womey	N6.430640° E2.295445°	
BE-Borgou	Badekperou	N9.211148 E2.556724	Soudano-Guinea savanna
	Bétérou	N9.198950 E2.265948	
	Boko	N9.414782 E2.618743	
	Bori	N9.755483 E2.407468	
	Bouyerou	N9.760339 E2.576970	
	Gamia	N10.339764 E2.719140	
	Gberouboue	N10.511914 E2.728443	
	Guessou	N10.129976 E2.650382	
	Ina	N9.977727 E2.723709	
	Ndali	N9.763031 E2.701058	
	Parakou town	N9.335888 E2.661661	
	Parakou range	N9.342134 E2.601775	
	Sanson	N9.274039 E2.421561	
	Sari river	N9.775620 E2.168167	
	Sérarou	N9.557771 E2.631839	
	Tchaourou	N8.841812 E2.601486	
BE-Collines	Agoua	N8.335842° E1.942079°	Soudano-Guinea savanna
	Bante	N8.462507° E1.841273°	
	Dassa	N7.728999° E2.188926°	
	Glazoue	N7.963816° E2.239028°	
	Gobe	N8.004876° E2.424571°	
	Gogoro	N8.270462° E2.645570°	
	Gouka	N8.153433° E1.968504°	
	Kokoro	N8.436247° E2.596481°	
	Logozohé	N7.888703° E2.097630°	
	Ouogi	N8.115906° E2.555405°	
	Paouignan	N7.632664° E2.206264°	
	Pira	N8.536243° E1.708091°	
	Savalou	N8.002668° E1.984564°	
	Toui	N8.646279° E2.591334°	

Table S1 (Continued)

Region	Locality	GPS coordinates	Ecological zone
BE-Couffo	Aplahoué	N6.978073° E1.658455°	Guinea savanna
	Daohoue	N7.055040° E1.813693°	
	Hagoumey	N6.846722° E1.760307°	
BE-Donga	Akékérou	N9.728982° E1.992160°	Soudano-Guinea savanna
	Aoro	N8.890072° E1.641903°	
	Basilla	N9.051283° E1.650603°	
	Batiaboto	N9.550660° E1.633067°	
	Bodi	N9.410646° E1.591996°	
	Diénendonanga	N9.728371° E1.807723°	
	Djougou	N9.687384° E1.682589°	
	Kodowari	N9.208293° E1.564968°	
	Kopargo	N9.836513° E1.548214°	
	Prékété	N8.725966° E1.644774°	
BE-Mono	Athiémé	N6.591010° E1.678187°	Guinea savanna
	Comè	N6.397313° E1.877987°	
	Gandjazoumé	N6.751284° E1.764917°	
	Grand Popo	N6.268668° E1.775946°	
	Hilacondji	N6.242640° E1.647031°	
	Sègbo	N6.526438° E1.817828°	
BE-Ouémé	Azové	N6.535082° E2.597178°	Guinea savanna
	Azaourissè	N6.680080° E2.501180°	
	Dagba	N6.818757° E2.471514°	
	Sémé-Kpodji	N6.387513° E2.623932°	
BE-Zou	Adjahakpa	N7.150800° E1.926796°	Guinea savanna
	Bohicon	N7.174440° E2.093613°	
	Dasso	N6.994215° E2.462461°	
	Doga	N7.229871° E2.430000°	
	Halagba	N7.018713° E2.189122°	
	Kpédékpo	N7.202996° E2.262586°	
	Massè	N7.137220° E2.513423°	
	Passagon	N7.316745° E2.080531°	
	Setto	N7.511448° E2.077530°	
	Zouto	N7.202996° E2.262586°	

*Sites where collections were regular and conducted properly to calculate parasitism rates
